# Complete Genome Sequence of *Mannheimia haemolytica* Strain Mh10517, Isolated from Sheep in South Africa

**DOI:** 10.1128/genomeA.00129-15

**Published:** 2015-04-09

**Authors:** Awoke Kidanemariam Gelaw, Wubetu Bihon, Ramagoma Faranani, Joseph Mafofo, Jasper Rees, Evelyn Madoroba

**Affiliations:** a Agricultural Research Council, Ondersteopoort Veterinary Institute, Ondersteopoort, South Africa; b Agricultural Research Council, Vegetable & Ornamental Plants Research Institute, Roodeplaat, South Africa; c Onderstepoort Biological Products, Ondersteopoort, South Africa; d Agricultural Research Council, Biotechnology Platform, Ondersteopoort, South Africa

## Abstract

Respiratory disease caused by *Mannheimia haemolytica* is a major concern in the cattle and small stock industry worldwide. This problem arises due to the interaction of numerous contributing factors, including physical stresses associated with weaning, shipment, inclement weather, and overcrowding coupled with viral and bacterial infections. The whole genome of *M. haemolytica* strain Mh10517 was analyzed using an Illumina MiSeq high-throughput sequencing platform. The genome size is 2.67 Mb with 2,879 predicted gene sequences. The availability of this genome sequence will advance studies on various aspects of the biology of *M. haemolytica* in Africa and the world at large.

## GENOME ANNOUNCEMENT

*Mannheimia haemolytica* is a facultative pathogen, a Gram-negative coccobacillus, and a commensal of the upper respiratory tract and nasopharynx of ruminant hosts (1, 2), but in the presence of stress factors, such as weaning, transportation, poor nutrition, and various viral infections, the bacterium multiplies and often results in fatal fibrinous pleuropneumonia, known as pneumonic pasteurellosis or shipping fever (3–10).

Genome sequences of *M. haemolytica* isolated from cattle (11) and sheep (10) in the United States have been determined and various putative genes with assigned functions were revealed. Here, we report the draft genome sequence of *M. haemolytica* strain Mh10517 isolated from the lung tissue of sheep with a history of respiratory infections in South Africa in 1966. To our knowledge, this will be the first genome sequence of *M. haemolytica* isolated from Africa.

Whole-genome sequencing of *M. haemolytica* strain Mh10517 was performed using an Illumina MiSeq sequencing platform. The chromosomal DNA was prepared as prescribed in the product manual. Briefly, *M. haemolytica* strain Mh10517 was grown overnight in brain heart infusion broth at 37°C. The next day, cells were harvested and genomic DNA was extracted using a DNeasy blood and tissue extraction kit (Qiagen) following the manufacturer’s instructions. Paired-end sequencing libraries with an average fragment length of 800 bp were constructed using a Nextera DNA sample preparation kit (Illumina). Sequencing was performed using the Illumina MiSeq sequencing platform, which produced a total of one million sequence reads with a length of 250 bases representing 100× coverage. The Illumina MiSeq sequencing platform produced 201,674 paired-end reads with an average length of 250 bp. The initial *de novo* assembly performed using CLC Genomics Workbench 6.0.1 produced 85 contigs.

The estimated genome size of *M. haemolytica* strain Mh10517 was 2.67 Mb with a G+C content of 40.9%, similar to those in previous reports (12, 13). Gene prediction and annotation conducted using the online RAST annotation server (14, 15) revealed a total of 2,879 predicted genes, including prophages. Of the 2,879 genes, 2,303 were protein encoding genes with assigned functions and 507 encoded hypothetical proteins, 19 rRNAs, 27 tRNAs, and 23 putative transposases.

### Nucleotide sequence accession numbers.

This whole-genome shotgun project has been deposited at DDBJ/EMBL/Genbank under the accession no. JPIZ00000000. The version described in this paper is version JPIZ01000000.
